# Depression in Patients With Parkinson’s Disease: A Hospital-Based Cross-Sectional Study

**DOI:** 10.7759/cureus.47214

**Published:** 2023-10-17

**Authors:** Priya Sujith, Porkodi Arjunan, Thomas Iype, Venkatesh Natarajan

**Affiliations:** 1 Medical Surgical Nursing, Government College of Nursing, Trivandrum, IND; 2 Nursing, Sri Ramachandra Institute of Higher Education and Research, Chennai, IND; 3 Medical Surgical Nursing, Sri Ramachandra Institute of Higher Education and Research, Chennai, IND; 4 Neurology, Government Medical College, Trivandrum, IND; 5 Physiotherapy, Sri Ramachandra Institute of Higher Education and Research, Chennai, IND

**Keywords:** tremor, bradykinesia, neuro psychiatric problems, non–motor symptoms, hoehn and yahr scale, hads, updrs, movement disorder, parkinson’s, depression

## Abstract

Introduction: Depression, a common non-motor symptom in Parkinson’s disease (PD), is often underdiagnosed and can significantly impact the quality of life (QOL) and treatment outcomes. Specific disease-related factors and non-specific factors may contribute to depression, and these factors should be identified early to plan the appropriate interventions that promote positive mood. The study aimed to assess the prevalence of depression in PD patients and to find out the factors associated with depression among patients with PD attending the neurology OPD of a tertiary care teaching hospital in Trivandrum.

Methods: A cross-sectional study was conducted at the neurology OPD of Government Medical College Hospital, Trivandrum, from December 2021 to February 2023. We included patients with PD diagnosed according to the United Kingdom PD Society Brain Bank criteria. We collected data from 220 patients with PD by interview technique. Hospital Anxiety and Depression Scale (HADS) was used to assess depression and anxiety in this study. Staging and the severity of the motor symptoms were assessed using the Hoehn and Yahr scale and the Movement Disorder Society Unified Parkinson’s Disease Rating Scale Part III (MDS UPDRS Part III), respectively.

Results: Among 220 patients with PD, 31.8% (95% CI: 4.36-5.40) had depression. The non-specific variables, such as education, living arrangements, and gender, and disease-specific variables, such as the severity of motor symptoms (MDS UPDRS Part III score) and the Hoehn and Yahr staging of PD, had a statistically significant association with depression. Logistic regression analysis showed that the severity of motor symptoms (OR=2.69, p=0.004)) and female gender (OR=1.830, p= 0.05) were the independent factors associated with depression.

Conclusion: Depression is a common non-motor symptom of PD that is often underdiagnosed and undertreated and can significantly impact the QOL of patients and their caregivers. Hence, it should be identified early and managed by pharmacological and non-pharmacological strategies.

## Introduction

Parkinson’s disease (PD) is the fastest-growing neurological disorder leading to disability and death. The aging population contributed too much to this growth. Globally, 6.1 million people have PD, with 3.2 million (52.5%) being males and 2.9 million (47.5%) being females. This number is projected to double to over 12 million by 2040 [1].

PD is a chronic progressive neurodegenerative disease of insidious onset that primarily affects movement leading to symptoms such as bradykinesia, rest tremor, rigidity, and postural disturbances. However, PD is not solely limited to motor symptoms; it can also have non-motor symptoms and become increasingly prevalent and obvious over the course of the disease. Non-motor features of PD are neuropsychiatric dysfunction, sleep disorders, autonomic dysfunction, sensory symptoms, and pain. Neuropsychiatric features include mood disorders such as apathy, anxiety, depression and anhedonia, cognitive dysfunction, and complex behavioral disorders [2-4].

Depression is one of the most common neuropsychiatric problems associated with PD. It is characterized by a state of low mood, sadness, hopelessness, and sometimes a feeling of emptiness or guilt [4]. Individuals with PD are at higher risk of developing depression than the general population. Studies have reported widely varying rates of depression among PD patients ranging from around 10% to as high as 60% [5-11]. The prevalence of depression in PD patients can vary depending on several factors, including the stage of disease, the specific population being studied, and the diagnostic criteria used. However, it is important to note that prevalence can vary in different populations and settings. Factors that contribute to depression in patients with PD are age, sex, history of depression or anxiety before PD diagnosis, severity of motor symptoms, disease duration, disease stage, levodopa dose, anxiety, sleep disturbances, and memory-related problems [5].

Depression in PD can significantly impact QOL, overall functioning, and treatment outcomes. It is often underdiagnosed and undertreated; thus, healthcare professionals must be vigilant in assessing and managing depression in PD. Early identification of depression and its associated factors will help to plan the interventions that promote positive mood and adjustments among people with PD [12]. This study aimed to assess the prevalence of depression in PD patients and to find out the factors associated with depression among patients with PD attending the neurology OPD of a tertiary care teaching hospital in Trivandrum.

## Materials and methods

This study has a cross-sectional analytical design. The study was conducted on 220 PD patients attending the neurology OPD of Government Medical College Hospital, Trivandrum, from December 2021 to February 2023. This design was selected to find out the factors associated with depression in PD patients. The sample size was calculated based on the prevalence rate of depression of 31.25% [13], precision of 80%, and standard value (Z) of 1.96. The total sample size was 220. Approval from the Institutional Ethics Committee (IEC-NI/19/NOV/71/83) and informed consent were obtained from the participants before data collection.

The samples who met the inclusion criteria were selected consecutively. We included both male and female adult patients with PD diagnosed according to the United Kingdom PD Society Brain Bank criteria and who are willing to participate in the study [14]. We excluded patients with severe visual/hearing impairment, severe dyskinesia, known cases of depression, and severe medical conditions like renal failure and liver failure.

The data was collected by interview technique. The socio-demographic data consisted of age, gender, education, marital status, religion, income status, type of family, living arrangements, and living area and the clinical data consisted of the duration of PD, history of smoking and alcoholism, treatment duration with levodopa, stage of PD, the severity of motor symptoms, and co-morbidities (diabetes mellitus, hypertension, heart disease, respiratory disease, and others). These data were collected using a structured questionnaire.

In addition to this, Hospital Anxiety and Depression Scale (HADS) was used to identify patients with depression and anxiety. HADS is a 14-item scale with seven items each for depression and anxiety. Scoring for each item ranges from 0 to 3. A score of >8 denotes anxiety or depression. The internal consistency and test-retest reliability of HADS are good in patients with PD. Cronbach alpha for HADS was 0.88 [15].

Staging of the PD was assessed using the Hoen and Yahr scale. There are five stages of PD progression from 1 to 5 [16]. Movement Disorder Society Unified Parkinson’s Disease Rating Scale Part III (MDS UPDRS Part III) was used to assess the severity of motor symptoms of PD. It consists of 18 items rated on a five-point Likert scale. The higher score reflects lower motor function. A score of zero is considered normal [17].

Statistical analysis

The data were analyzed using SPSS Statistics version 20.0 (IBM Corp. Released 2011. IBM SPSS Statistics for Windows, Version 20.0. Armonk, NY: IBM Corp.). Descriptive statistics (e.g., mean, standard deviation, percentage, and 95% CI) and inferential statistics (chi-square test and binary logistic regression) were used to find out the association between the variables of interest. Binary logistic regression analysis was done for the variables that showed univariate association (p<0.1). A value of p<0.05 is considered statistically significant.

## Results

We presented the socio-demographic characteristics of 220 patients with PD. Among them, 50.5% were males. The mean age of patients with PD was 60.36 (7.86). Sixty percent of patients belonged to the Hindu religion. Widows/widowers constituted 11.8%, single 3.2%, and separated 1.4%. One hundred forty patients (63.6%) were in the above-poverty line (APL) category and the remaining 36.4% were in the below-poverty line (BPL) category. Half of the patients with PD had 8-10 years of formal education. About 81.8% of patients were living with their spouse. Among 210 patients, 43.6% had no co-morbidities and 90% had no history of PD in their family. A third of patients with PD had a disease duration of >6 years. The mean disease and treatment duration with levodopa among PD patients were 5.0 (3.03) and 4.4 (2.8), respectively. Half of the patients with PD (49.5%) belonged to Hoehn and Yahr stage 2. The mean score of MDS UPDRS Part III was 32.35 (17.1). Half of the participants had an MDS UPDRS Part III score of <30 (Table 1).

**Table 1:** Socio-demographic and clinical characteristics of participants PD: Parkinson’s disease, MDS UPDRS Part III: Movement Disorder Society Unified Parkinson’s Disease Rating Scale Part III, APL: above-poverty line, BPL: below-poverty line, HTN: hypertension

Variables		Frequency	Percentage
Age in years	40-49	23	10.5
	50-59	67	30.5
	60-69	105	47.7
	>69	25	11.4
Gender	Male	111	50.5
	Female	109	49.5
Marital status	Married	184	83.6
	Single	7	3.2
	Widow/widower	26	11.8
	Separated	3	1.4
Religion	Hindu	132	60
	Christian	52	23.6
	Muslim	36	16.4
Education in years	No formal education	12	5.5
	1-4	22	10
	5-7	32	14.6
	8-10	110	50
	10-12	22	10
	>12	22	10
Income status	APL	140	63.6
	BPL	80	36.4
Living arrangement	Spouse	180	81.8
	Children	33	15
	Alone	5	2.3
	Others	2	0.9
Family history of PD	Yes	22	10.0
	No	198	90.0
Smoking	Current smoker	5	2.3
	Never a smoker	203	92.3
	Ex-smoker	12	5.5
Alcoholism	Current alcoholic	2	9
	Never alcoholic	212	96.4
	Ex-alcoholic	6	2.7
Co-morbidities	Absent	96	43.6
	Diabetes	24	10.9
	Hypertension	59	26.8
	Heart disease	4	1.8
	Respiratory disease	3	1.4
	Both diabetes and HTN	24	10.9
	Others	10	4.6
Duration of PD in years	1	22	10
	1-3	52	23.6
	3-6	65	29.5
	≥6	67	30.5
Stage of PD	1	42	19.1
	2	109	49.5
	3	58	26.4
	4	9	4.1
	5	2	0.9
MDS UPDRS Part III score	<30	112	50.9
	≥30	108	49.1

Prevalence of depression in PD patients

Table 2 shows that the prevalence of depression among patients with PD was 31.8% (95% CI: 4.36-5.40). The mean HADS score for depression was 4.88 (3.92).

**Table 2:** Distribution of participants based on the HADS score for depression HADS: Hospital Anxiety and Depression Scale

HADS score	Depression			95% CI
	Frequency	Percentage	Mean (SD)	
0-7 (normal)	150	68.2	4.88 (3.92)	
8-21 (depression)	70	31.8		4.36-5.40

Table 3 shows that 21.8% of patients with PD had anxiety (95% CI: 4.51-5.41) and the mean anxiety score was 4.96 (3.39).

**Table 3:** Distribution of participants based on the HADS score for anxiety HADS: Hospital Anxiety and Depression Scale

HADS score	Anxiety			95% CI
	Frequency	Percentage	Mean (SD)	
0-7 (normal)	172	78.2	4.96 (3.39)	
8-21 (anxiety)	48	21.8		4.51-5.41

Association between socio-demographic and clinical characteristics of participants and depression

The variables, such as education (p<0.05), living arrangements (p=0.05), gender, and marital status (p=0.07), and disease-specific variables, such as the severity of motor symptoms (MDS UPDRS Part III score) and Hoehn and Yahr staging of PD (p<0.001), had a statistically significant association with depression in univariate analysis (Table 4).

**Table 4:** Association between socio-demographic and clinical characteristics and depression * Included in the logistic regression analysis (p<0.1) MDS UPDRS Part III: Movement Disorder Society Unified Parkinson’s Disease Rating Scale Part III, PD: Parkinson’s disease, APL: above-poverty line, BPL: below-poverty line

Variables		Depression		ᵪ^2^	p
		Yes	No		
Age in years	≤61	40	78	0.51	0.48
	>61	30	72		
Gender	Male	29	82	0.35	0.07*
	Female	41	68		
Marital status	Single	4	3	5.1	0.07*
	Married	53	131		
	Others	13	16		
Education	<10 years	62	114	4.7	0.03*
	≥10 years	8	36		
Living area	Rural	61	116	2.9	0.09*
	Urban	9	34		
Income status	APL	43	97	0.22	0.64
	BPL	27	53		
Living arrangement	Spouse	51	129	5.91	0.05*
	Children	15	18		
	Others	4	3		
Comorbidities	No	32	64	0.18	0.67
	Yes	38	86		
MDS UPDRS Part III Score	<30	22	90	15.6	0.001*
	≥30	48	60		
Hoehn and Yahr staging	<3 years	36	115	14.2	0.001*
	≥3 years	34	35		
Duration of PD	<4 years	33	84	1.50	0.22
	≥4 years	37	64		

Factors associated with depression using binary logistic regression

Table 5 shows that female gender (OR=1.830, P= 0.05) and MDS UPDRS Part III score >30 (OR=2.69, P=0.004) were the independent factors associated with depression.

**Table 5:** Factors associated with depression using binary logistic regression MDS UPDRS Part III: Movement Disorder Society Unified Parkinson’s Disease Rating Scale Part III

Variables	OR	95% CI	p
Gender	1.830	1.00-3.50	0.05*
Education	0.56	0.23-1.36	0.20
Living arrangement	1.47	0.80-2.72	0.22
MDS UPDRS Part III score	2.69	1.38-5.25	0.004*
Hoehn and Yahr staging	1.62	0.82-3.17	0.16

## Discussion

Depression is the most common non-motor symptom associated with PD. PD patients were significantly more depressed than any other medical and surgical conditions [18]. Every patient with PD experienced non-motor symptoms and 90% had more than five non-motor symptoms. Of that, 46% experienced depression as a non-motor symptom [10]. Another study reported that 86% of patients with PD had neuropsychiatric features and 28% had depression. It has a detrimental effect on QOL and results in caregiver burden [4]. The QOL was significantly worse in PD patients with depression than without depression in the physical and psychological domains. Early evaluation and treatment of non-motor symptoms in PD potentially improve QOL and reduce morbidity and healthcare costs [13]. The present study assessed depression and its associated factors in 220 PD patients.

In the current study, the prevalence of depression in PD patients was 31.8% (95% CI: 4.36-5.40). These findings were in line with other existing literature where 28.8% of patients with PD had depression [4]. Like our study, Khedr et al. reported the prevalence as 31.25% [13]. The prevalence of depression among PD patients varies from 10% to 60% [4-11,19]. These variations may be due to inconsistencies in sampling procedures, assessment techniques, and definitions given to depression.

In the present study, 21.8% of PD patients had anxiety. This was supported by Cui et al. where the prevalence was 25.81% and that anxiety disorder and sleep disorder are the two factors that significantly affected depression in PD patients [6].

In the present study, the factors associated with depression in univariate analysis were education (p<0.05), living arrangement (p=0.05), gender (p=0.07), stage of PD (p<0.001), and severity of motor symptoms assessed by MDS UPDRS Part III score (p<0.001). Like Cui et al., Khedr et al. also reported that depression was associated with advanced disease stage and higher MDS UPDRS Part III scores [6,13]. Rissardo et al. reported that depression was associated with lower education levels [20]. Regarding living arrangements, a study done in an older adult population in China reported that compared with living only with a spouse, people living with a spouse and child or living alone were more likely to have depressive symptoms [21]. Some studies reported that depression and anxiety were more common among patients with a longer duration of PD [6], but, in our study, there is no relationship existing between the duration of PD and depression.

Logistic regression analysis was done for the variables that showed univariate association (p<0.1) and found that female gender (OR=1.830 and CI: 1.00-3.50) and severity of motor symptom MDS UPDRS Part III score >30 (OR=2.69 and CI: 1.38-5.25) were the significant risk factors associated with depression. These findings were compared with the findings by Khedr et al., where depression was higher among females and total MDS UPDRS Part III and Hoehn and Yahr scale accounted for 33.4% of the variance for depression. Advanced disease stage and severity were independent predictors of depression [13].

There are a few limitations in our study that should be considered when interpreting the results. The present study has its weakness in being a cross-sectional sectional study. Hence, a well-defined longitudinal study methodology will improve the power of the study. The prevalence of depression in PD patients in the present study was 31.8% (95% CI: 4.36-5.40). In the present study, depression was measured using HADS instead of the Diagnostic and Statistical Manual of Mental Disorders, Fifth Edition, criteria which is the gold standard for depressive disorders. However, the internal consistency and test-retest reliability of the HADS are good in patients with PD. Cronbach alpha for the HADS was 0.88. Hence, it can be used to screen depression in PD patients [22,23]. Another limitation is that there were only a few patients in the advanced stage of PD (stages 4 and 5), which may not represent the entire PD population since our study was conducted in the neurology OPD only.

## Conclusions

Depression is a common non-motor symptom of PD that is often underdiagnosed and undertreated. The present study aimed to recognize and address depression in PD patients to improve their overall well-being. Results suggest that the frequency of depression is high, and it is a prevalent non-motor symptom in PD patients. The severity of motor symptoms, female gender, advanced stage of PD, and low education level were significantly associated with depression. The study highlights the need for improved recognition and treatment of depression in PD patients. Apart from motor symptoms, there is an unmet need to address non-motor symptoms to improve the QOL and to reduce the caregiver burden. Hence, it should be identified early and managed by pharmacological and non-pharmacological strategies.
